# Anti-Melanogenic Mechanism of Tetrahydrocurcumin and Enhancing Its Topical Delivery Efficacy Using a Lecithin-Based Nanoemulsion

**DOI:** 10.3390/pharmaceutics13081185

**Published:** 2021-07-31

**Authors:** Xudong Tang, Qiaoru Dong, Jun Li, Fang Li, Bozena B. Michniak-Kohn, Denggao Zhao, Chi-Tang Ho, Qingrong Huang

**Affiliations:** 1 Department of Food Science, Rutgers University, 65 Dudley Road, New Brunswick, NJ 08901, USA; xt51@scarletmail.rutgers.edu (X.T.); qd44@scarletmail.rutgers.edu (Q.D.); ctho@sebs.rutgers.edu (C.-T.H.); 2 College of Food Science, South China Agricultural University, Guangzhou 510642, China; 249885990@scau.edu.cn; 3 College of Food Science and Engineering, Wuhan Polytechnic University, Wuhan 430023, China; LF12149@whpu.edu.cn; 4 Center of Dermal Research (CDR) and Ernest Mario School of Pharmacy, Life Sciences Building, Rutgers University, Piscataway, NJ 08854, USA; michniak@pharmacy.rutgers.edu; 5 School of Biotechnology and Health Sciences, Wuyi University, Jiangmen 529020, China; zhaodenggao@wyu.edu.cn

**Keywords:** tetrahydrocurcumin, B16F10, HaCaT, topical delivery, anti-melanogenesis, nanoemulsion

## Abstract

Tetrahydrocurcumin (THC) has been well known for its superior antioxidant properties. Therefore, it is speculated that it might be effective to relieve oxidative stress-induced diseases, such as skin hyperpigmentation. In this work, an in vitro B16F10 melanoma cell model was used to study the impact of THC on the melanogenic process under stressed conditions. It was demonstrated that THC could effectively inhibit the α-MSH (melanocyte-stimulating hormone) induced melanin production in B16F10 melanoma cells and the expressions of three key enzymes involved with the biosynthetic process of melanin, tyrosinase (TYR), tyrosinase-related protein 1 (TRP-1), and tyrosinase-related protein 2 (TRP-2), were all significantly reduced. In addition, an in vitro human keratinocyte cell model was used to investigate the potential protective role of THC on H_2_O_2_-induced cytotoxicity. It was found that THC could prevent H_2_O_2_-induced oxidative stress based on the results of both the cell viability study and the intracellular ROS (reactive oxygen species) study assessed by the flow cytometry. Last, THC was formulated into a lecithin based nanoemulsion, and an in vitro Franz diffusion cell study using Strat-M^®^ membrane concluded that the nanoemulsion could significantly enhance the membrane permeation compared to the unformatted THC suspension. This research demonstrated the anti-melanogenic benefits of THC on the melanoma and keratinocyte cell models and the topical delivery efficacy could be significantly enhanced using a lecithin based nanoemulsion.

## 1. Introduction

The skin is the largest organ in the human body, and its main functions are to regulate the moisture content and the temperature within the body, provide protection from external microorganisms, ultraviolet (UV) radiation, chemicals, etc. [1,2]. The epidermis is the outermost layer of the skin [3], approximately 90% of the epidermal cells are keratinocytes, which are responsible for the skin barrier function and are also involved in many biochemical reactions [3,4,5,6,7]. When the skin is exposed to external stress such as UV radiation, reactive oxidative species (ROS) are generated and accumulate extensively in the skin, which is usually detrimental to the cells [8]. For example, ROS such as H_2_O_2_ could permeate through the nuclear membrane in the keratinocytes and react with DNA-associated transition metals to generate hydroxyl radicals and cause DNA base damage [9,10,11,12]. In addition, high levels of ROS could also directly affect melanocytes, which regulate enzymatic activity, and cause unpleasant skin hyperpigmentation that can even lead to the initiation and progression of melanoma, a deadly skin cancer. Additionally, UV radiation could stimulate the synthesis of certain biochemical factors such as alpha-melanocyte-stimulating hormone (α-MSH), endothelin-1 (ET-1), stem cell factor (SCF), etc. These then could be secreted from keratinocytes and delivered to the neighboring melanocytes [13,14]. When melanocytes receive the excessive biochemical factors from keratinocytes, they upregulate the enzymes responsible for melanin synthesis and cause the overproduction of melanin, leading to skin hyperpigmentation.

Tetrahydrocurcumin (1,7-bis(4-hydroxy-3-methoxyphenyl)heptane-3,5-dione) is a major metabolite of curcumin, a polyphenolic compound derived from the rhizome of the herb *Curcuma longa* L [15]. Many studies have demonstrated THC’s superior antioxidant properties and its potential role as a chemopreventive agent for a variety of diseases [16,17,18]. Based on the antioxidant properties of this compound, THC is probably able to mitigate the oxidative stress in the skin caused by UV radiation and other sources. In addition, some studies also reported other skin-related benefits of THC such as acceleration of wound healing by reducing the epithelialization time as well as promotion of collagen production in the dermis, etc. [19,20,21].

Though there are many benefits of THC in a variety of areas, the delivery of THC to the skin is limited by its poor aqueous solubility. Lecithin derived formulations are frequently used today to enhance the topical delivery efficacy, such as liposomes and nanoemulsions. This is due to the fact that lecithin has the unique advantages of high biodegradability, similar structure to the lipid layer under the skin, and low irritancy to the skin [22,23,24]. In addition, lecithin is also known for its moisturizing effect. Liposome has the capacity to deliver both hydrophilic and hydrophobic compounds, but it does have limitations such as poor stability and drug loading [23]. On the contrary, lecithin based nanoemulsions have high solubilizing capacity and better stability, and thus are commonly used to facilitate the topical delivery of lipophilic compounds [23]. It exhibits a fluidic microstructure and high skin affinity [25,26,27]. Another advantage of nanoemulsions is the absence of organic solvents during preparation, which makes it a preferable candidate as a drug delivery system.

In this study, the protective role of THC on the reduction of H_2_O_2_-induced oxidative stress in HaCaT cells was first examined, then THC’s impacts on the generation of melanin in an α-MSH induced B16F10 melanoma cell model and its mechanism were explored. Diethylene glycol monoethyl ether (trade name: Transcutol^®^) and medium chain triglycerides (MCT) were used in the lecithin based nanoemulsions to optimize the solubility capacity and permeation-enhancing properties of the vehicle within the skin. The topical delivery efficacy was evaluated using an in vitro Franz diffusion cell model. The results of this work will provide insights about the anti-melanogenic benefits of THC and its future potential topical application in the pharmaceutical and cosmetic industries.

## 2. Materials and Methods

### 2.1. Materials

Tetrahydrocurcumin (THC) was a gift from Sabinsa (East Windsor, NJ, USA). Diethylene glycol ethyl ether (DEGEE, trade name: Transcutol^®^) was a kind gift from Gattefossè (Paramus, NJ, USA). Medium chain triglyceride (MCT) was obtained from Stephan (Northfield, IL, USA). Lipoid S 75 (Lecithin, Tocopherol; Contains 70% phosphatidylcholine) was a kind gift from American lecithin (Oxford, CT, USA). Methanol and Acetonitrile were purchased from Sigma-Aldrich (St. Louis, MO, USA). Milli-Q water (18.3 MΩ) was used in all experiments. HaCaT human keratinocytes were obtained from the Kunming cell bank, the Chinese Academy of Sciences (Kunming, China). Hydrogen peroxide solution (30%, *v*/*v*) was purchased from Shhushi (Shanghai, China). B16F10 melanoma cells were purchased from ATCC (Manassas, VA, USA). α-MSH and l-3,4-dihydroxyphenylalanine (l-DOPA) were purchased from Yuanye Biotechnology (Shanghai, China). 3-(4,5-dimethylthiazol-2-yl)-2,5-diphenyltetrazolium bromide (MTT) was purchased from Sigma (St. Louis, MO, USA).

### 2.2. HaCaT Cell Culture and Cell Viability Studies

HaCaT cells were cultured in Dulbecco’s Modified Eagle’s Medium (DMEM) supplemented with 10% (*v*/*v*) fetal bovine serum, 4 mM glutamine, 1% (*v*/*v*) penicillin, and 1% (*v*/*v*) streptomycin (Hyclone Laboratories, Logan, UT, USA) in a 37 °C humidified incubator with 5% CO_2_ air.

Following treatment with different concentrations (0, 0.5, 1, 2, and 4 µg/mL) of THC (in dimethyl sulfoxide (DMSO) and diluted with culture medium, final DMSO concentration < 0.4%), the cell viability was evaluated using the MTT colorimetric assay [28]. Briefly, HaCaT cells were seeded at a density of 1 × 10^4^ per well in a 96-well microtiter plate, they were cultured with selected concentrations of THC in the medium for 24 h, respectively. Later, the medium was removed, the cells were washed with phosphate buffered saline (PBS), MTT solution (0.5 mg/mL) was added, and then it was incubated for 4–6 h. Lastly, the medium was discarded and 150 µL DMSO was added in order to dissolve the formazan crystals. The absorbance of each well was recorded at 490 nm using a microplate reader (Envision 2105, Perkin Elmer, Waltham, MA, USA). Cell viability was calculated using the following equation (1). 0.4% DMSO with cells was used as the control group and 0.4% DMSO as the blank.

(1) $$Cell\;viability\;\%=\frac{OD_{sample}-OD_{blank}}{OD_{control}-OD_{blank}}\times 100\%$$

where *OD* is the optical density.

To evaluate the effects of hydrogen peroxide induced oxidative stress on the cell viability, cells (1 × 10^4^ cells per well) were cultured with different concentrations of hydrogen peroxide (150, 300, 400, and 600 µM) for 24 h. Later, the medium was again removed and the cells were washed with PBS and incubated with MTT solution for another 4–6 h. Finally, the medium was discarded and 150 µL DMSO was added. Cell viability was subsequently analyzed using a microplate reader and Equation (1) was used to determine the cell viability.

To evaluate the potential protective effects of THC on the H_2_O_2_ induced cell cytotoxicity, cells (1 × 10^4^ cells per well) were cultured with selected concentrations of THC (0, 0.5, 1, 2, and 4 µg/mL) for 2–3 h first. Then, the medium was removed, different concentrations of hydrogen peroxide (150, 300, 400, and 600 µM) were added, and the cells were cultured for another 24 h. Later, the medium was again removed, the cells were washed with PBS, and then they were incubated with MTT solution for another 4–6 h. Finally, the medium was discarded and 150 µL DMSO was added. Cell viability was subsequently analyzed using a microplate reader and Equation (1) was used to determine the cell viability.

### 2.3. Measurement of Intracellular ROS in HaCaT

Cells (2 × 10^5^/well) were seeded in a 6-well plate and cultured using the method described above. Selected concentrations of THC (0.5, 1, 2, and 4 µg/mL) were added to the cells and cultured for another 12 h. Subsequently, 600 µM hydrogen peroxide solution was added to the cells and incubated for one hour to induce oxidative stress. Then, 10 µM 2′-7′dichlorofluorescin diacetate (DCFH-DA) was added to the cells and the cells were incubated for 1 h. The fluorescence strength of the cells was measured using flow cytometry (Beckman Coulter, Indianapolis, IN, USA) with an excitation wavelength of 490 nm and an emission wavelength of 530 nm, respectively. The peroxide levels in the cells were plotted as one-parameter histograms with cell count on the *y*-axis and fluorescence on the *x*-axis.

### 2.4. Measurement of α-MSH

HaCaT cells were cultured following the same method described in Section 2.3. First cells were pretreated with selected concentrations of THC, then 600 µM hydrogen peroxide solution was added to the cells and incubated for another hour. α-MSH levels in the HaCaT supernatant were determined using an ELISA kit (Shanghai Ding Biological Tech, Shanghai, China) following the manufacturer’s instructions.

### 2.5. B16F10 Cell Culture and Cytotoxicity Assay

Cells were cultured in DMEM, supplemented with 10% (*v*/*v*) fetal bovine serum, 1% (*v*/*v*) penicillin, and 1% (*v*/*v*) streptomycin at 37 °C in a humidified incubator at 5% CO_2_. Cells were seeded in a 96-well plate at a density of 1 × 10^4^ cells per well and then cultured for 48 h with selected concentrations of THC (0, 0.05, 0.5, 1, 10, 20, and 40 µg/mL in DMSO and diluted with culture medium, final DMSO concentration <0.4%). MTT solution was then added, and the cells were cultured for another 4 h. Later, the medium was discarded and 150 µL DMSO was added. Finally, the absorbance of each well was measured at a wavelength of 490 nm using a microplate reader. Cell viability was calculated using Equation (1).

### 2.6. Measurement of Melanin Content in B16F10 Cells

Measurement of melanin content in the B16F10 cells was determined based on the published method with slight modifications [29]. Briefly, cells were seeded at a density of 1 × 10^4^ cells per well in a 96-well plate, then cells were treated with two different concentrations of THC (1 µg/mL, 10 µg/mL) and α-MSH (0.5 µM). Untreated cells, which were free of THC and α-MSH, acted as the control. Cells treated with α-MSH only were the negative control. After 48 h, all the groups of cells were washed with PBS, harvested, lysed, and the melanin was solubilized in 1 N NaOH (including 10% DMSO) at 80 °C for 1 h. Melanin content was determined by measuring absorbance at 475 nm using a microplate reader.

### 2.7. Measurement of Tyrosinase Activity in the B16F10 Cells

Cells were seeded at a density of 10^6^ cells per well in a 6-well plate. They were treated with 2 mL of 10 µg/mL THC and 0.5 µM α-MSH, or free of THC but with 0.5 µM α-MSH, or free of THC and α-MSH for 48 h, respectively. After incubation, the medium was removed and cells were washed twice with PBS. Then, cells were detached and centrifuged at 1000 rpm for 5 min. Later, cell pellets were re-suspended in PBS containing 1% (*v*/*v*) Triton X-100 (with 1 µg/mL Leupeptin, 100 µg/mL phenylmethylsulfonyl fluoride) at 4 °C. Then, the suspension was centrifuged at 12,000 rpm for 10 min. The supernatant (90 μL) was collected, mixed with 10 μL of l-DOPA solution, and incubated for 20 min at 37 °C. Absorbance was measured at 475 nm using a microplate reader. Tyrosinase activity was expressed as the percentage of the control and was normalized by the total protein contents using the enhanced BCA protein assay kit (Beyotime, Shanghai, China) [30].

### 2.8. Real-Time Polymerase Chain Reaction (RT-PCR)

Total RNA was extracted from cells using the TaKaRa MiniBEST Universal RNA Extraction Kit (TaKaRa, Japan) following the manufacturer’s instructions. cDNA was then synthesized using PrimeScript RT reagent kit with gDNA eraser (Takara, Japan).

RT-PCR was performed using a CFX Connect Real-Time PCR detection system (BIO-RAD, USA). Lightcycler^®^ 480 SYBR Green I Master (Roche, Penzberg, Germany) was used for amplification and detection of DNA. The qRT-PCR primer sets for tyrosinase, TRP1, and TRP2 were purchased from Applied Biosystems (Foster City, CA, USA) as summarized in Table 1, and target gene expression was normalized to glyceraldehyde-3-phosphate dehydrogenase (GAPDH). Relative quantization was performed using the comparative ∆∆Ct method according to the manufacturer’s instructions.

### 2.9. Western Blot Analysis

B16F10 cells were seeded at a density of 10^6^ per well in a six-well plate and pre-treated without α-MSH as the control, with 0.5 µM α-MSH, or with both 0.5 µM α-MSH and 10 µg/mL THC as the test sample. After 48 h, the cells were washed with PBS, detached, lysed and harvested on cold ice. Then, cell lysates were centrifuged at 12,000 rpm for five mins at 4 °C and the supernatant was collected. The total protein content was determined by the BCA protein assay kit (P0009, Beyotime, Shanghai, China). Then, equal amounts of protein were separated by 12% SDS-PAGE (sodium dodecyl sulfate-polyacrylamide gel electrophoresis) and transferred to a polyvinylidene difluoride (PVDF) membrane. The membrane was later incubated with primary antibodies including anti-tyrosinase antibody (AF5491, Affinity Biosciences, Cincinnati, OH, USA), anti-TRP-1 antibody (ab178676, ABCAM, Cambridge, MA, USA), anti-TRP-2 antibody (Proteintech Group, Wuhan, China), and GAPDH polyclonal antibody (ATPA00013Rb, Atagenix, Wuhan, China) at 4 °C overnight. Finally, it was incubated with secondary antibodies, goat anti-mouse IgG (H+L), HRP conjugate (SA00001-1, Proteintech Group, Wuhan, China), goat anti-rabbit IgG (H+L), and HRP conjugate (SA00001-2, Proteintech Group, Wuhan, China) for 1 h at room temperature. Protein bands were visualized using an enhanced chemiluminescence (ECL) kit (Yitong Biotech, Wuhan, China) and analyzed using the ChemiDocTMXRS+ imaging system (Bio-Rad, Hercules, CA, USA). The quantification of relative protein expressions, i.e., grey values, were carried out using Image Lab 3.0 software and GAPDH as an internal standard.

### 2.10. Determination of THC’s Solubility in Selected Solvents

The solvents MCT, argan oil, sunflower oil, and DEGEE were selected and their individual capacity to solubilize THC was evaluated using the following methods. An excess quantity of THC (700 mg) was added to 7 mL of each individual solvent and mixed for 24 h using a magnetic stirrer at room temperature. Then, the 1.5 mL samples were centrifuged at 9000 rpm for 10 min. The supernatant was collected, filtered through a 0.45 µm filter, and diluted with methanol. Then, the THC’s concentration in each solvent was determined by the method described below in Section 2.14 in order to determine the solubility in each vehicle.

### 2.11. Fabrication of the Nanoemulsion

Briefly, THC (1% *w*/*v*) was first solubilized in the oil phase including Transcutol and MCT oil at 50–60 °C. Once THC was fully solubilized, then the oil phase was added to the water phase containing 4.72% (*w*/*v*) lecithin. Two separated phases were kept mixing under magnetic stirring for 10–15 min until homogeneity was reached. The coarse emulsion was formed using a high-speed homogenizer (Ultra-Turrax T25, IKA Works Inc., Wilmington, NC, USA) at 10,000 rpm for 1–3 min. Finally, the nanoemulsion was formed through an EmulsiFlex-C6 high-pressure homogenizer (AVESTIN Inc., Ottawa, ON, Canada) at 120 MPa for a total of three passes.

### 2.12. Particle Size Measurements, LUMiSizer^®^ Test & Polarized Microscopy of the Nanoemulsions

The nanoemulsions were centrifuged under the stressed condition using LUMiSizer^®^, the instability index was obtained by the SEPView^®^ software (Version 6.0, LUM GmbH, Berlin, German) by analyzing the change in the transmission profiles over time. This is an efficient method for the fast screening of the formulations [31]. Then, the particle size of the nanoemulsion was measured by a dynamic light scattering mode-based particle size analyzer (Model 90 Plus, Brookhaven Instrument Corp., Holtsville, NY, USA) at a fixed scattering angle of 90⁰ and room temperature. Each time, 50 µL of the nanoemulsion was diluted to 25 mL distilled water. The nanoemulsion was also characterized by polarized light miscopy (Leica DFC 550, Wetzlar, Germany). Both techniques as well as the visual observation of the samples were conducted in order to monitor the stability of the nanoemulsions. Samples were stored at two different conditions, including room temperature for four weeks and 50 °C for one week.

### 2.13. In Vitro Franz Diffusion Cell Permeation Study

Franz diffusion cells (FDC-6, Logan Instrument Corp., NJ, US) with a diffusion area of 1.32 cm^2^ were used to study and comparisons were made of the permeation rates using THC in the nanoemulsions vs. the THC suspension. Briefly, 1.5 mL sample (contained the equivalent 1% *w/v* THC content) was loaded in the donor, 8 mL pH 6.8 PBS (50% *v/v* ethanol) was filled in the receptor. The solubility of THC in the receptor medium was around 5 mg/mL, which could ensure sink conditions during the study. Strat-M^®^ membrane was mounted between the donor and receptor medium. The temperature was maintained at 37 ± 0.5 °C. 1 mL samples were collected from the receptor at fixed time intervals (0.5, 1, 2, 4, 6, 8, 12, and 24 h) for HPLC analysis, and then the receptor medium was replaced with fresh medium. To assess the retention of THC with the Strat-M^®^ membrane, the membrane was collected at the end of 24 h. It was rinsed with pH 6.8 PBS free of ethanol twice front and back, then it was dried with a cotton swab. Later, the membrane was extracted with 5 mL methanol solution for 5–10 min. Lastly, the quantity of the retention could be determined by the mass within the membrane divided by the surface area, which is 1.32 cm^2^.

All the experiments were replicated in triplicate. Steady state flux (J_ss_, μg/h∙cm^2^) was calculated by the linear regression analysis from the slope of cumulative THC permeated per cm^2^ against the length of time.

The cumulative permeation Q and permeation enhancement ratio (ER) were calculated using Equations (2) and (3) [32]:

Q (μg/cm^2^) = (∑M)/A (2)

ER = (Q (THC in nanoemulsion))/(Q(THC suspension in water))(3)

where M represents the mass (g) of THC found in the receptor and A represents the surface area of permeation, which in this case is equal to 1.32 cm^2^.

### 2.14. High Performance Liquid Chromatography (HPLC)

The quantitative analysis of THC in the sample was determined using Agilent 1100 series HPLC (Santa Clara, CA, USA) with UV detector (280 nm). A Phenomenex C18 column (5 µm, 4.6 × 150 mm) was used. The mobile phase consisted of 50% *v/v* acetonitrile and 50% *v/v* water (including 0.1% *v/v* formic Acid). The column was eluted under isocratic condition and the flow rate is 1.0 mL/min. The injection volume was 20 μL. The linearity measurements were performed using a series of serial dilutions ranging from 6.25 to 100 μg/mL with an R^2^ value of 0.9925. The limit of quantification (LOQ) was 2.5 μg/mL.

### 2.15. Statistical Analysis

Data are expressed as means ± SD (standard deviation) and analyzed with one-way ANOVA. A *p*-value < 0.05 was considered statistically significant.

## 3. Results

### 3.1. MTT Assay-HaCaT

**Figure 1:**
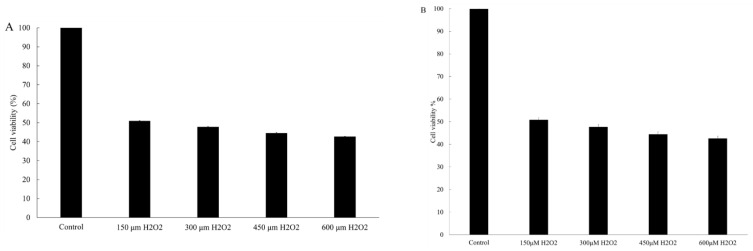
Effect of various concentrations of tetrahydrocurcumin (THC) (**A**) and H_2_O_2_ (**B**) on the viability of HaCaT cells after 24 h of treatment.

The various concentrations of THC that were tested did not elicit any cytotoxic effect on the HaCaT cells (Figure 1A). Separately, a series of concentrations of H_2_O_2_ (150, 300, 450, and 600 μM) were added to induce oxidative stress (Figure 1B). It was concluded that H_2_O_2_-induced damage was concentration dependent. The viability at 150, 300, 450, and 600 μM H_2_O_2_ was reduced to 50.7%, 47.9%, 45.2%, and 42.6%, respectively.

**Figure 2:**
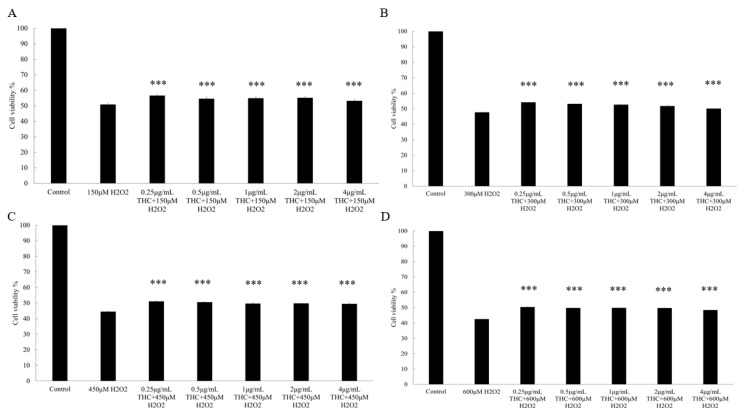
Effect of various tetrahydrocurcumin (free of THC as the control group, 0.5, 1, 2, and 4 μg/mL) concentrations and each individual H_2_O_2_ concentration (150 (**A**), 300 (**B**), 450 (**C**), and 600 (**D**) μM) on the viability of HaCaT cells after 24 h. Results are expressed as mean SD (*n* = 3). *** *p* < 0.001 compared to the cells treated with H_2_O_2_ only in each group.

In addition, when the cells were pretreated with THC prior to H_2_O_2_, it was demonstrated that THC could protect the cells from the H_2_O_2_ induced cell death (Figure 2). When varied concentrations of THC were added to the respective 150 μM (A), 300 μM (B), 450 μM (C), and 600 μM (D) H_2_O_2_ group, THC could increase the cell viability and such effects were not dose dependent.

### 3.2. Measurement of Intracellular ROS in HaCaT and α-MSH

**Figure 3:**
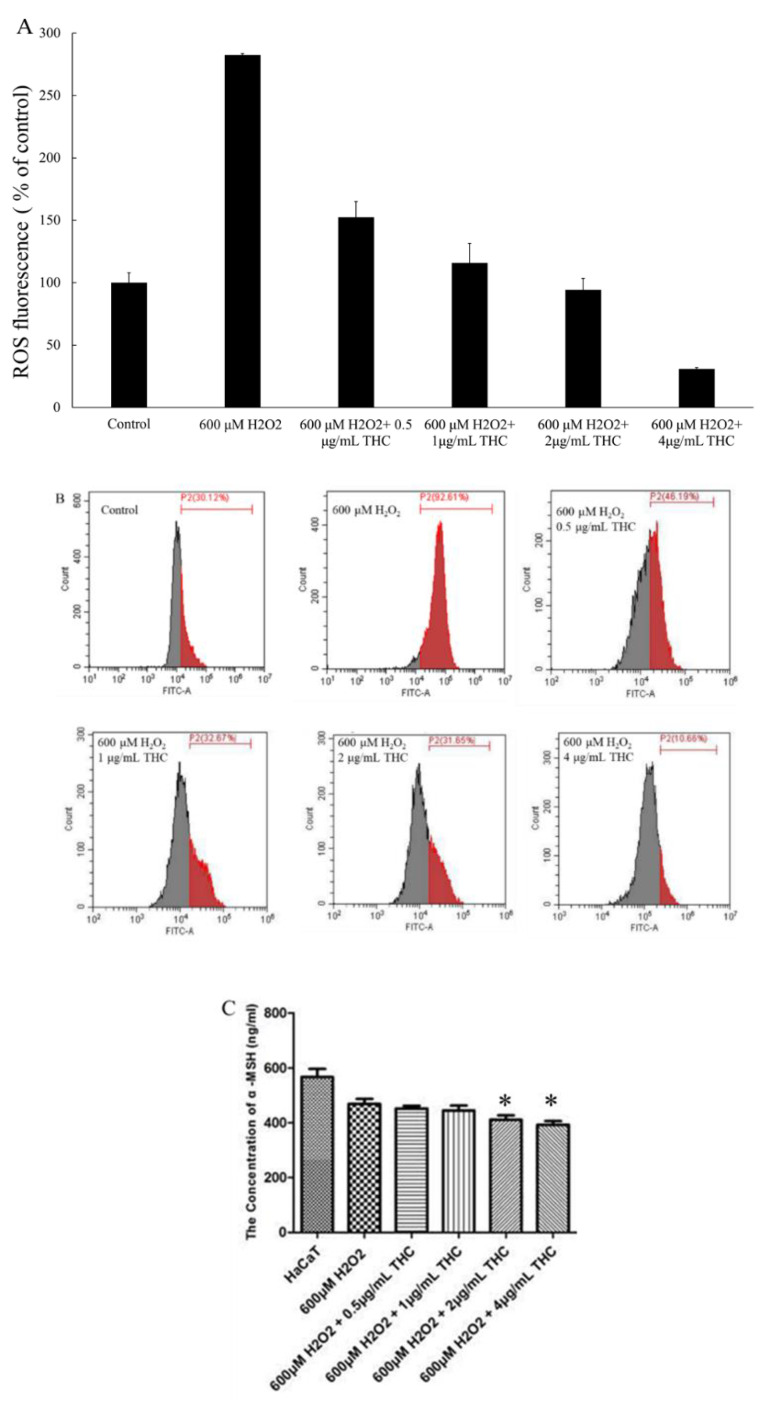
Protective role of THC on the H_2_O_2_ induced ROS levels in HaCaT. (**A**) The summary of ROS levels in HaCaT cells (**B**) Intracellular ROS levels of HaCaT were assessed by flow cytometry. (**C**) α-MSH levels were found in the supernatant in HaCaT, after the cells were pretreated without or with the selected concentrations of THC (0.5, 1, 2, and 4 μg/mL) for 12 h prior to the addition of H_2_O_2_. Results are expressed as mean SD (*n* = 3). * *p* < 0.05 compared to the cells treated with 600 μM H_2_O_2_ only.

In order to further study the protective role of THC in terms of relieving the oxidative stress in HaCaT, intracellular ROS generation was quantified with the aid of DCFH-DA as the probe. Fluorescence intensity, which represented the ROS level within the cell, was measured using the flow cytometry and the result was presented in Figure 3A,B. As shown, in the presence of 600 μM H_2_O_2_, ROS level was observed to increase significantly, almost three times higher compared to the cells without the treatment of H_2_O_2_. When THC was added prior to the H_2_O_2_ treatment, it protected the cells by reducing the ROS levels directly and the effect was concentration dependent. Specifically, when different THC concentrations (0.5, 1, 2, 4 μg/mL) were added prior to the H_2_O_2_ treatment, ROS level (% of control) was observed to decreased to 152.5%, 115.7%, 94.4%, and 30.7%, respectively. This provided evidence that pretreatment with THC is effective at protecting the cells from H_2_O_2_ induced ROS attacks. Figure 3C demonstrated that THC (2, 4 μg/mL) was able to reduce the production of α-MSH in the HaCaT cells under oxidative stress compared to the group treated with H_2_O_2_ only.

### 3.3. MTT Assay-B16F10

**Figure 4:**
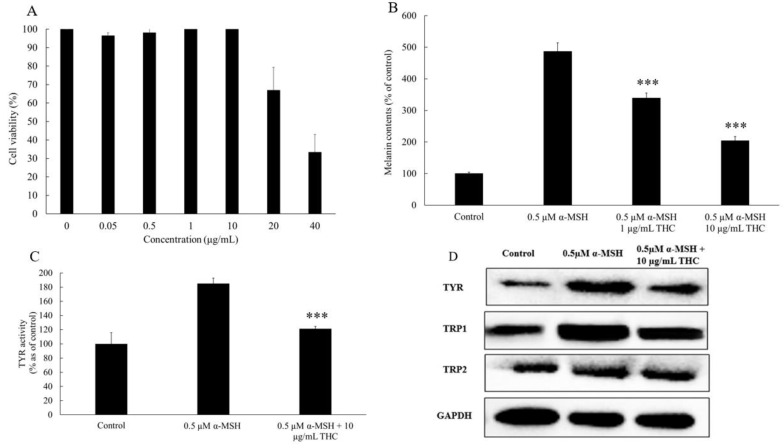
Anti-melanogenic effect of THC on the B16F10 cells. (**A**) Effects of THC on the viability of B16F10 melanoma cells after 48 h treatment. (**B**) Melanin content comparison between control group, cells treated with 0.5 μM MSH, and 0.5 μM α-MSH with 1 μg/mL and 10 μg/mL THC, respectively after B16F10 cells were cultured for 48 h. (**C**) Cellular tyrosinase (TYR) activity. (**D**) Western blotting analysis of protein expressions for TYR, tyrosinase related protein 1 (TRP1), tyrosinase related protein 2 (TRP2), and Glyceraldehyde 3-phosphate dehydrogenase (GAPDH) as the internal reference in B16F10. Results are expressed as mean SD (*n* = 3). *** *p* < 0.001 compared to group treated with 0.5 μM α-MSH.

Melanogenesis is an overall oxidative process, therefore the use of an antioxidant is considered a potential treatment to control the melanogenic process, especially after or during oxidative stress [33]. In order to further understand the anti-melanogenic mechanism of THC, the B16F10 melanoma model was used. Cell viability was evaluated using the MTT assay method, and it concluded that THC had no significant cytotoxic effect on B16F10 cell viability at or below 10 µg/mL. As indicated in Figure 4A, cell viability was reduced to 66.0% when THC was added at 20 µg/mL and 33.4% when THC was added at 4 µg/mL. Based on this result, concentrations of 1 µg/mL and 10 µg/mL were selected for the subsequent experiments.

### 3.4. Melanin Content and Cellular Tyrosinase Activity Assay in B16F10 Cell Model

α-MSH is responsible for regulating the production of melanin [34]. In this work, it is used to stimulate melanin production. When 0.5 μM α-MSH was added to B16F10 cells, melanin production increased significantly, it was approximately five times higher compared to the control group (free of α-MSH) as shown in Figure 4B. When THC (1 μg/mL, 10 μg/mL) was added and co-cultured with α-MSH. It was found that THC was effective in inhibiting the production of α-MSH induced melanin in B16F10 cells. Separately, proteins were extracted from the cells and mixed with l-dopa. The reaction intensity of l-dopa to dopaquinone within the fixed time (20 min) was used to quantify the tyrosinase activity. α-MSH increased the tyrosinase activity to 184.7% compared to the control and 10 μg/mL THC could inhibit such an effect, decreasing the activity down to 120.9% (Figure 4C). In order to further understand the mechanism of THC’s impact on the melanogenic process, protein expression and mRNA levels were studied, and the results are summarized in the following sections.

### 3.5. Western Blot Analysis

The result from the Western blot was summarized in Figure 4D and the grey values from the protein bands were quantified with the aid of Image Lab 3.0 software (Table 2). As indicated, THC inhibited the expressions of all the key enzymes involved in the α-MSH induced melanogenesis including tyrosinase, TRP-1, and TRP-2. This explains why THC is able to inhibit the α-MSH induced melanin production as well as the tyrosinase activity (data reported in the previous section).

### 3.6. Realtime Polymerase Chain Reaction

**Figure 5:**
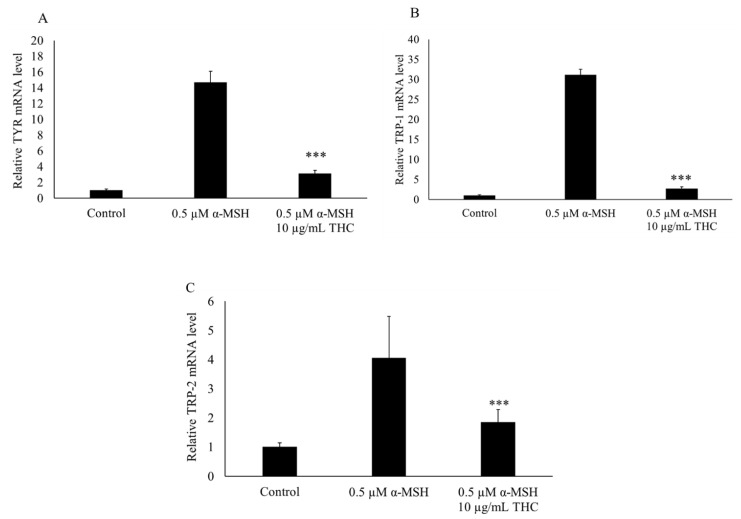
Effects of THC on mRNA expression of genes for TYR (**A**), TRP-1 (**B**), and TRP-2 (**C**) in B16F10 melanoma cells. Levels of mRNA were determined by PCR and GAPDH was used as the internal reference. Results are expressed as mean SD (*n* = 3). *** *p* < 0.001 compared to group treated with α-MSH.

As shown in Figure 5, after 48 h, THC was found to reduce the mRNA levels for TYR, TRP-1, and TRP-2.

### 3.7. Determination of THC’s Solubility in Selected Solvents

**Figure 6:**
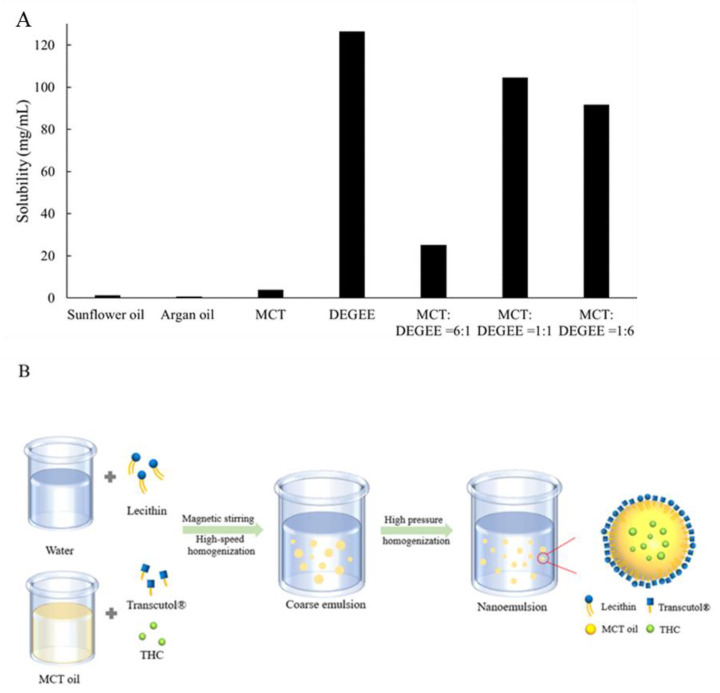
THC’s solubility in medium chain triglyceride (MCT), sunflower oil, argan oil, diethylene glycol monomethyl ether (DEGEE), or combination of MCT and DEGEE at various volumetric ratios (1:1, 1:6, 6:1) (**A**). Schematic illustration for preparing the lecithin based nanoemulsion (**B**).

THC is crystalline in nature, and it has been reported to have very limited aqueous solubility, approximately 56 ng/mL, and crystallinity of the active ingredient is often the main cause for the low permeation through the skin [20,35,36,37]. Therefore, it is critical to enhance the compound solubility first in order to maximize its delivery through the skin. A few solvents were selected, including DEGEE-hydrophilic permeation enhancer, and a group of oils that with various carbon chain lengths, content of the unsaturated fatty acids, etc. The capacity of each solvent to solubilize THC was evaluated, the results are summarized in Figure 6A. It was found that DEGEE showed the highest solubility for THC (approximately 126.4 mg/mL), 14 times higher compared to MCT (around 9.4 mg/mL). Sunflower oil had a solubility of 1.2 mg/mL, and argan oil had the lowest solubility, which was around 0.6 mg/mL. Based on these results, a combination of MCT and DEGEE at different ratios were prepared, each of their solubility capacities was evaluated using the same method. It has been reported before that the combination of binary solvents could promote skin permeation though a mechanism that is not well known yet [38]. Therefore, the combination of DEGEE and MCT was selected to be used for the formulation of the nanoemulsion.

### 3.8. Stability Study for the Lecithin Based Nanoemulsions

LUMiSizer^®^ was first used to evaluate the stability of the different formulations under stressed conditions. This technology is based on the record for the changes of the samples’ transmission profile at constant or variable centrifugal force after a certain period. Instability index is usually calculated and used to predict the emulsion stability [31]. It can be obtained by dividing the increase in transmission (initiation of the separation induced by emulsion creaming, sedimentation, etc.), which indicates the separation at a certain time, by the maximum clarification (complete separation).

As summarized in Table 3, when the lecithin dispersion decreased to 30% or lower, a higher instability index (from phase separation) was observed, indicating the critical role of lecithin as the emulsifier. Based on this result, new formulations were proposed and used for the subsequent experiment (Table 4).

**Figure 7:**
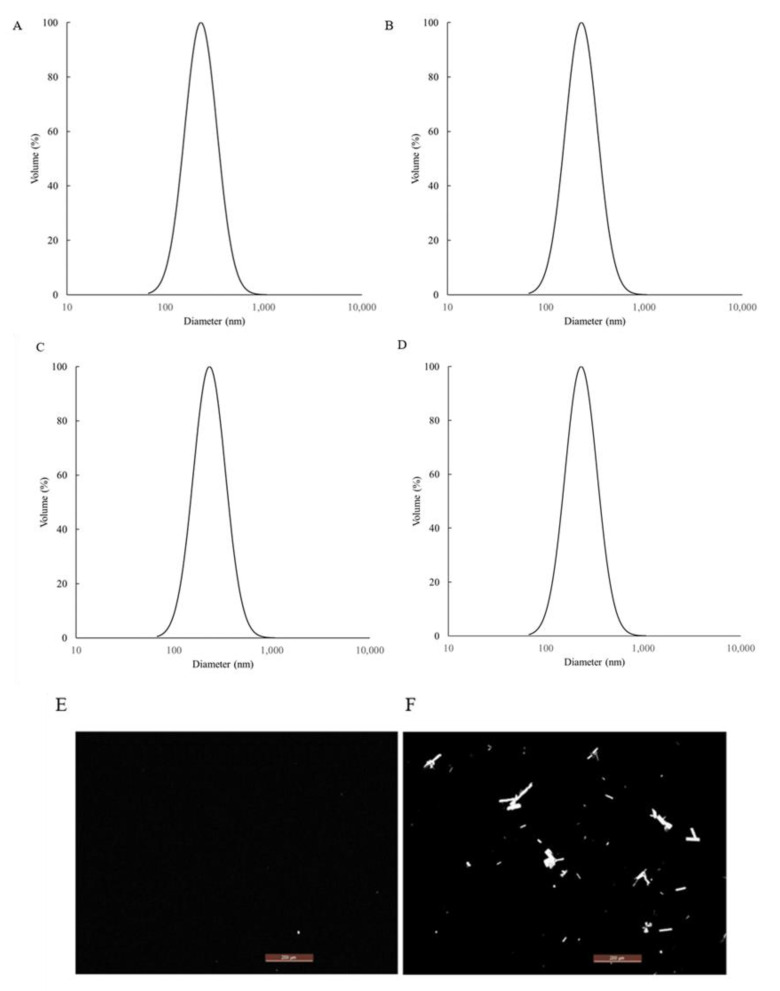
Particle size results of nanoemulsion F1 at time zero (**A**); 1 week at 50 °C (**B**); 1 week at room temperature (**C**); four4 weeks at room temperature (**D**). Comparison of the polarized microscope results after 1 week at 50 °C between nanoemulsion formula F1 (**E**) and F2 (**F**).

The nanoemulsion F1 and micellar solution F2 (absence of MCT oil phase) were produced using the steps summarized in Figure 6B, and the stability of the samples was monitored at both 50 °C and room temperature. Polarized light microscopy, particle size measurement, as well as visual inspection were the main methods used to evaluate the stability. From the polarized microscope results (Figure 7), it was observed that THC started to recrystallize in F2 quickly after one week at 50 °C. Comparing F1 and F2, it showed that the presence of MCT was critical to preventing the recrystallization of THC. Additionally, particle size measurements were also monitored, and the results were also summarized in Figure 7. As the data indicated, nanoemulsion F1 was stable with no noticeable particle size change. Visual inspection was also conducted, which confirmed that there was no visible phase separation, oil agglomeration, etc., for F1 at the end of one month under both storage conditions.

### 3.9. In Vitro Franz Diffusion Cell Study

**Figure 8:**
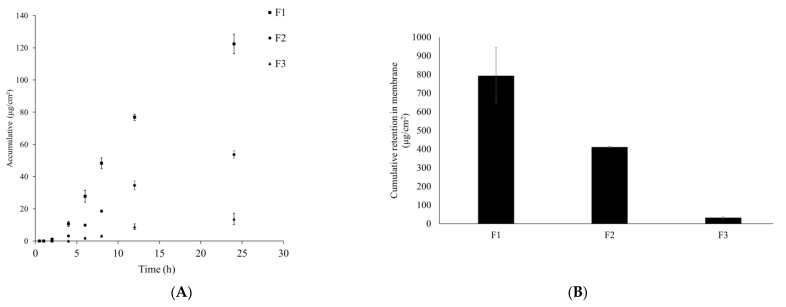
Comparison of in vitro permeation results of THC between nanoemulsion F1, micellar solution F2, and THC suspension (**A**). Comparison of the cumulative retention of THC in the membrane after 24 h (**B**).

Nanoemulsion F1 and micellar solution F2 showed a significant permeation enhancement compared to F3, the THC suspension (Figure 8). At the end of the 24 h experiment, enhancement ratios for F1 and F2 were 9.3 and 6.5, respectively. In addition, the steady state flux for F1, F2, and F3 (THC suspension in water) was 5.6, 3.9, and 0.6 μg/cm^2^/h. Both results demonstrated that the nanoemulsion F1 was a successful formulation with a capacity to enhance the permeation of the active ingredient. In addition, when comparing the results between F1 and F2, a higher percentage of MCT oil in the formulation seemed to promote the permeation as well as enhance the overall stability preventing THC from recrystallization. Hypothetically, the slightly higher permeation ER from F1 could be explained by the potential lower solubility for THC in the binary solvents (MCT and DEGEE at 3:1, *v*/*v*), which led to a higher thermodynamic activity of THC in the system and higher cumulative permeation rate was observed as a result [39]. It is also possible that the binary solvents including MCT and DEGEE, when combined at a specific ratio (3:1, *v*/*v*), produced a synergistic, permeation enhancing effect. Nevertheless, F1 is considered to be a successful formulation for the topical delivery enhancement and the schematic illustration about its enhancing permeation through the skin is presented in Figure 8B. Since THC is lipophilic and has a partition coefficient log P value of 2.98 [20], it has a high permeation rate. However, THC is crystalline in nature, which explains the phenomenon of the relatively low permeation rate for the THC water suspension. On the other hand, when THC was solubilized and delivered through the nanoemulsion, the enhancing permeation rate could be explained due to the combination of the factors including the change from crystalline to amorphous status for THC and the permeation enhancement from the delivery system.

## 4. Discussion

In the first part of the study, we used an in vitro HaCaT cell model to investigate the protective role of THC on the oxidative stress-induced attack. H_2_O_2_ was used to induce oxidative stress, which could inhibit the cell proliferation, biological macromolecular damage, cell apoptosis, etc., [40]. In reality, the increasing levels of H_2_O_2_ in the epidermis could be produced directly from the environmental factors such as UVB and air pollution. The neutral nature of H_2_O_2_ would enable it to permeate through the membrane and diffuse from between the cells causing the damage [41]. As shown in Figure 1B, the viability of HaCaT cells decreased after exposure to different levels of H_2_O_2_, and such effects were concentration dependent when THC concentrations of 0.5, 1, 2, and 4 μg/mL were added prior to H_2_O_2_ exposure. THC was found to protect the HaCaT cells from the H_2_O_2_-induced cytotoxicity to different degrees. However, THC’s protective effects were not significantly high based on the MTT results. In order to ensure that THC became accessible to the cells, the incubation time was optimized in the following ROS study. It was concluded that THC could significantly reduce the ROS level within the cells. In addition, α-MSH level was also reduced when THC was added prior to the H_2_O_2_ compared to the group treated with H_2_O_2_ only. As α-MSH is known to upregulate the enzymes responsible for melanin synthesis [13,14], THC’s antioxidant role might help to mitigate the hyperpigmentation through that pathway as well. Another interesting point was that once H_2_O_2_ was added, lower α-MSH levels were observed. This was due to the fact that α-MSH was oxidized by the H_2_O_2_, which changed the binding capacity with the antibody in the ELISA kit, reducing the levels [42]. In conclusion, pretreatment of THC could be an effective method to protect HaCaT from attacks induced by oxidative stress. One explanation could be that the hydrogenation structure in THC enables a strong hydrogen-donating ability leading to the high antioxidant property [43]. Another explanation could be that THC upregulates the superoxide dismutase, glutathione, etc., to enhance the defense against oxidative stress [44]. Secondly, an in vitro B16F10 melanoma cell study was conducted and it was concluded that THC could decrease the activity of tyrosinase, which is the key enzyme responsible for melanin synthesis. In addition, THC could also inhibit the gene expressions of the three major enzymes that are involved in the melanin biosynthetic process, including tyrosinase, TRP-1, and TRP-2. The mRNA expression levels for TYR, TRP-1, and TRP-2 were measured by a real-time polymerase chain reaction (RT-PCR) in order to further understand the mechanism of THC on the suppression of α-MSH induced melanogenesis. THC reduced all three relevant mRNA expression levels. To summarize, the data using the B16F10 model demonstrated the anti-melanogenic role of THC. This result is consistent with previously published works [45], which concluded that THC was able to decrease the α-MSH induced melanin production even at a low concentration (0.1 μg/mL). Therefore, THC might be efficient to control or reduce the melanin contents by its inhibitory impacts on the enzyme’s activity, or its impacts on the production of α-MSH under oxidative stress based on its superior anti-oxidant property. In the future, it would also be interesting to conduct an interaction study between the two cells (keratinocytes and melanocytes) to further understand THC’s role in terms of anti-melanogenesis.

Next, lecithin was used as the emulsifier to fabricate the nanoemulsion. It is known that it can interact with the lipid bilayer in the stratum corneum and be retained in this layer, which makes it a great candidate for topical delivery [46,47]. Many studies have demonstrated that lecithin based nanoemulsion is an excellent platform for the topical delivery [23,48]. In our study, we first prepared a series of formulations with various lecithin levels and/or oil levels and then we used LUMiSizer^®^ to quickly screen the formulations based on the accelerated stability result [31]. LUMiSizer^®^ results demonstrated the critical role of lecithin in terms of emulsion stability. Formula F1 was developed based on the stability result and the hypothesis that the oil phase in the nanoemulsion would promote the retention of the formulation within the skin. As the solubility and stability results indicated, though the addition of MCT did not contribute to a higher solubility compared to DEGEE as the neat solvent, the hydrophobic MCT in the lecithin-based nanoemulsion could provide a preferrable environment for the lipophilic THC when dispersed into water. DEGEE, on the other hand, is a hydrophilic ingredient that has showen a higher solubility for the compound when used as a neat solvent; however, its hydrophilic nature is not favorable to THC and might lead to recrystallization. An in vitro Franz diffusion model with a synthetic membrane, comparable to human cadaver skin [49], was later used to evaluate and compare the permeation performance. Lecithin-based nanoemulsion F1 demonstrated the highest skin permeation performance and retention compared to micellar solution F2 (freshly prepared before the permeation study to minimize the impact from recrystallization found in Section 3.8) and the THC suspension. There are several mechanisms, including interaction with the stratum corneum lipid organization, providing skin hydration, and adherence to the skin or synthetic membrane, that could help to explain this phenomenon [50,51]. The result from this study demonstrated the application of lecithin-based nanoemulsions for topical drug delivery enhancement.

## 5. Conclusions

We first demonstrated the protective role of THC for keratinocytes under oxidative stress. Later, THC’s anti-melanogenic role was studied and confirmed using the B16F10 and keratinocytes cell lines. The data obtained provided evidence about the benefits of THC on skin. We developed a nanoemulsion and used an in vitro Franz diffusion cell model to evaluate the topical delivery efficacy. Compared to the unformulated THC suspension, the nanoemulsion could significantly enhance the delivery efficacy approximately seven-fold. In conclusion, this work demonstrated that THC could work as an effective anti-melanogenic agent and that the lecithin based nanoemulsion could significantly enhance the topical delivery efficacy.

## Figures and Tables

**Table 1:** The primer sequences used in the real-time polymerase chain reaction measurement for GAPDH (glyceraldehyde 3-phosphate dehydrogenase, internal standard), tyrosinase, tyrosinase related protein-1 (trp-1), and tyrosinase related proten-2 (trp-2).

GAPDH	Forward	5′-TGTTTCCTCGTCCCGTAGA-3′
	Reverse	5′-GATGGCAACAATCTCCACTTTG-3′
Tyrosinase	Forward	5′-TCCAAGAGTCAGATCCAGGC-3′
	Reverse	5′-TCCTGGGGTTGCTTCTTCTT-3′
TRP1	Forward	5′-AGTGGCTGCGTTGTTACTTG-3′
	Reverse	5′-TGGAGTGGTTAGGATTCGGG-3′
TRP2	Forward	5′-CTTTTGGACCATGTTCGGCA-3′
	Reverse	5′-AGGAGTTGGTGATCATGGCA-3′

**Table 2:** Quantitative results of the grey values from the Western blot analysis. Grey values for tyrosinase (**A**); trp1 (**B**); trp2 (**C**). GAPDH acted as the internal standard.

**A**			
Group	tyrosinase	GAPDH	tyrosinase: GAPDH
Control	343144	1310392	0.262
0.5 µM α-MSH	1057134	1097892	0.963
0.5 µM α-MSH + 10 µg/mL THC	641184	1157389	0.554
**B**			
Group	trp1	GAPDH	trp1: GAPDH
Control	705731	1310392	0.539
0.5 µM α-MSH	1695207	1097892	1.544
0.5 µM α-MSH + 10 µg/mL THC	1088834	1157389	0.941
**C**			
Group	trp2	GAPDH	trp2: GAPDH
Control	620056	1310392	0.473
0.5 µM α-MSH	910823	1097892	0.829
0.5 µM α-MSH + 10 µg/mL THC	687519	1157389	0.594

**Table 3:** Compositions of the formulations for initial screening and the results of the instability index from LUMiSizer^®^.

Ingredients/ID	S1	S2	S3	S4	S5	S6	S7
8% (*w*/*w*) Lecithin dispersion	20%	30%	40%	50%	50%	50%	50%
Water	49%	39%	29%	19%	9%	--	29%
DEGEE	10%	10%	10%	10%	10%	10%	10%
THC	1%	1%	1%	1%	1%	1%	1%
MCT	20%	20%	20%	20%	30%	39%	10%
Instability index	0.746	0.411	0.053	0.049	0.061	0.062	0.023

**Table 4:** Compositions of the formulations F1, F2, and F3.

Ingredients	F1	F2	F3
(8% *w*/*v*) Lecithin dispersion	50%	50%	--
Medium Chain Triglyceride	30%	--	--
DEGEE	10%	10%	--
THC	1%	1%	1%
Water	9%	39%	99%
Instability index	0.061	0.018	N/A *

* Suspension has not been tested on LUMiSizer^®^ before. N/A means not available.

## Data Availability

The data presented in this study are available from the corresponding author.
